# The Effect of Nanoemulsion as a Carrier of Hydrophilic Compound for Transdermal Delivery

**DOI:** 10.1371/journal.pone.0102850

**Published:** 2014-07-28

**Authors:** Ming-Jun Tsai, Yaw-Syan Fu, Yu-Hsuan Lin, Yaw-Bin Huang, Pao-Chu Wu

**Affiliations:** 1 Department of Neurology, China Medical University Hospital, School of Medicine, Medical College, China Medical University, Taichung, Taiwan, ROC; 2 Department of Neurology, Tainan Municipal An-Nan Hospital, Tainan, Taiwan, ROC; 3 Department of Biomedical Science and Environmental Biology, Kaohsiung Medical University, Kaohsiung, Taiwan, ROC; 4 School of Pharmacy, Kaohsiung Medical University, Kaohsiung, Taiwan, ROC; RMIT University, Australia

## Abstract

The purpose of the present study was to investigate the effect of nanoemulsions as a carrier vehicle of hydrophilic drug for transdermal delivery. The response surface methodology with a mixture design was used to evaluate the effect of ingredient levels of nanoemulsion formulations including cosurfactant (isopropyl alcohol, 20∼30%), surfactant (mixed of Brij 30 and Brij 35, 20∼30%), and distilled-water (34.5∼50.0%) on properties of the drug-loaded nanoemulsions including physicochemical characters and drug permeability through rat skin. The result showed that the hydrophilic drug in aqueous solution with or without penetration enhancer could not transport across rat skin after 12 h of application. Used nanoemulsions as carrier vehicle, the permeation rate of drug was significantly increased from 0 to 63.23 µg/cm^2^/h and the lag time was shortened from more than 12 h to about 2.7∼4.0 h. Moreover, the drug-loaded nanoemulsion formulation also showed physicochemical stability after 3 month storage at 25°C and 40°C.

## Introduction

Transdermal drug delivery offers many benefits over other traditional routes of administration including non-invasiveness, accessibility, avoidance of first-pass metabolism, compliance, ease of drug input termination in problematic cases, and controllable drug delivery rates [1], [2]. Ropinirole hydrochloride (RHCl) is a selective non-ergoline dopamine D2 receptor agonist, which can stimulate striatal dopamine receptors to produce dopamine, hence it has been prescribed for Parkinson's disease treatment [3], [4]. It is a potent drug with a dose of 2 mg to be administered 3–4 times daily. In view of the characteristics of RHCl including small oral dosage (3–9 mg daily), low molecular weight (MW = 260), short elimination half-life (about 6 h), and low bioavailability (approximately 50%) because of extensive first-pass metabolism, oral administration is problematic [3]–[6]. In additional, dysphagia is a frequent and potentially serious complication of Parkinson's disease [7]. RHCl seems to be a good candidate for transdermal administration; hence, it was used as a model drug in this study.

The greatest obstacle for drug transdermal delivery is the barrier property of stratum corneum, a 10 µm to 20 µm thick tissue layer composed of a structured lipid/protein matrix [8], [9]. Numerous strategies including used carrier vehicle, chemical penetration enhancers, and physical technologies such as electroporation, iontophoresis, ultrasound, and microneedle technologies either singly or in combination, have been used to facilitate the permeability of therapeutical compounds through the skin [10]–[13]. A previous study [14] pointed out that small droplet size provides a better chance for adherence to biological membranes transporting therapeutic compounds in a controlled manner. Hence, nano- or micro- carriers such as ethosomes, nanoemulsions, liposomes, and polymeric nanoparticles have been widely used to improve permeability of therapeutic agents through skin in recent years [1], [15]–[17]. Nanoemulsion is an isotropic and thermodynamically stable colloidal system with a mean droplet size in range of 10–100 nm [18]. In general, a nanoemulsion formulation contains the four major ingredients of water, oil, surfactant, and cosurfactant. The surfactant is used to decrease the interfacial tension between oil and aqueous phase, and then form a nanoemulsion. Cosurfactant could provide further decrease in interfacial tension and to fluidize the interfacial surfactant film. Moreover, it can decrease the used amount of surfactant in nanoemulsion preparation and influence the drug-loaded nanoemulsion transportation through the skin [19], [20]. The four ingredients may decrease the diffusion barrier of the skin by acting as penetration enhancer [20]. Many studies have reported that using nanoemulsion as vehicle can enhance the transportation of drug through the skin over conventional topical products such as ointments, gels, and creams [21]–[24]. Furthermore, nanoemulsions can be manufactured by a spontaneous emulsifying method which provides some advantages over other carriers such as polymeric nanoparticles and liposome, including low cost preparation procedure, high hydrophilic and lipophilic drug loading, and long shelf life for therapeutic agents [25]–[27]. Hence, the nanoemulsions were used as the vehicle to facilitate the transportation rate of the hydrophilic model drug RHCl through rat skin in this study.

## Materials and Methods

### Materials

Ropinirole hydrochloride (RHCl) was purchased from Glenmark Generics Limited (Mumbai, India). Polyoxyl 23 lauryl ether (Brij 35) and Polyoxyl 4 lauryl ether (Brij 30) were from Acros Organic (Pennsylvania, USA). Sorbitan monolaurate (Span 20, HLB = 8.6) was from Tokyo Chemical Industry (Tokyo, Japan). Polyoxyethylene sorbitan monooleate (Tween 80, HLB = 15) was acquired from Showa Corporation (Saitama, Japan). Isopropyl myristate (IPM) and isopropyl alcohol (IPA) were purchased from Merck Chemicals (Darmstadt, Germany). Caffeine was from Wako Pure Chemical Industries Ltd, (Tokyo, Japan). All other chemicals and solvents were of analytical reagent grade.

### Preparation of drug-loaded Nanoemulsions

The RHCl-loaded nanoemulsions were prepared by spontaneous emulsion method. The mixture surfactants were mixed well in advance. Oil phase (IPM) was mixed thoroughly with mixture surfactants and cosurfactant by a vortex at room temperature. Subsequently, distilled water was slowly added to the mixture and mixed evenly with a vortex. After the clarity and transparency of blank nanoemulsions were formed, RHCl was dissolved in the blank nanoemulsions by a shaker for 10 min. There was no precipitate observed in the final RHCl-loaded nanoemulsion.

In order to estimate the degree of effects of the formulation factors and obtain an optimal formulation, the response surface methodology [28]–[30] was applied in this study. RHCl of 0.5% nanoemulsions were prepared. According to preliminary study, the amount of IPM was fixed at 5% because RHCl is a hydrophilic compound (133 mg/m L), and the amount of oil phase can't influence the solubility. The other level of ingredients in nanoemulsions such as cosurfactant of IPA (20∼30%), mixture surfactant of Brij 30 and Brij 35 (20∼30%), and double-distilled water (34.5∼50.0%) were selected as formulation variables. The ranges of formulation variables were set according to our preliminary study. The total amount of the three ingredients was fixed at 95% of total amount of nanoemulsion formulation. Ten model RHCl-loaded nanoemulsions were arranged randomly, based on the constrained mixture model (Design-Expert software). The compositions of RHCl-loaded nanoemulsions are listed in Table 1.

**Table 1:** The composition, physicochemical properties, and permeability parameters of RHCl-loaded nanoemulsions.

	MS %	CoS %	IPM %	Drug %	Size (nm)			Viscosity (cps×10^3^)			Flux (µg/cm^2^/h)			LT (h)		
F1	20B	30E	5	0.5	14.73	±	1.16	8.99	±	0.12	44.21	±	5.73	4.3	±	1.2
F2	25B	30E	5	0.5	12.73	±	1.50	9.71	±	0.11	48.53	±	5.16	3.0	±	0.0
F3	30B	30E	5	0.5	21.03	±	7.60	9.79	±	0.07	25.94	±	3.51	4.3	±	0.6
F4	30B	20E	5	0.5	14.10	±	0.66	19.10	±	0.56	20.25	±	4.23	5.3	±	0.6
F5	20B	30I	5	0.5	14.40	±	2.79	6.67.	±	0.04	65.45	±	13.2	3.7	±	0.6
F6	30B	20I	5	0.5	12.67	±	0.81	13.33.	±	0.06	30.61	±	2.71	3.7	±	0.6
F7	15T	30E	5	0.5	107.93	±	7.14	11.37	±	0.21	13.08	±	0.57	4.0	±	0.0

RHCl: ropinirole hydrochloride, MS: Mixture surfactant, CoS: Cosurfactant, IPM: Isopropyl myristate,

B: Brij30/Brij35 (4/1); T: Tween80/Span20 (2/3), E: Ethanol, I: Isopropyl alcohol.

LT: lag time

#### Nanoemulsion characterization

Viscosities of drug-loaded nanoemulsions were determined in triplicate using a cone-plate of viscometer (Brookfield, Model LVDV-II, USA). A sample of 0.5 mL was placed in the plate, the temperature of which was maintained at 37°C by thermostatic pump for 3 mins. The rotation rate of viscometer was set at 120 rpm. The viscosity value was recorded 20 s after measurement had begun.

Mean droplet size and droplet size distribution of RHCl-loaded nanoemulsions were measured by a photo correlation spectroscopy equipped with laser light scattering (Zetasizer 3000HSA, Malvern, UK). The intensity of the light scattering was observed at a fixed angle of 90°. The helium-neon laser of λ was set at 633 nm. A sample of 3 mL was loaded in a cuvette and placed in the scattering chamber to measure the mean droplet size and droplet size distribution.

### Skin permeation study

**Figure 1:**
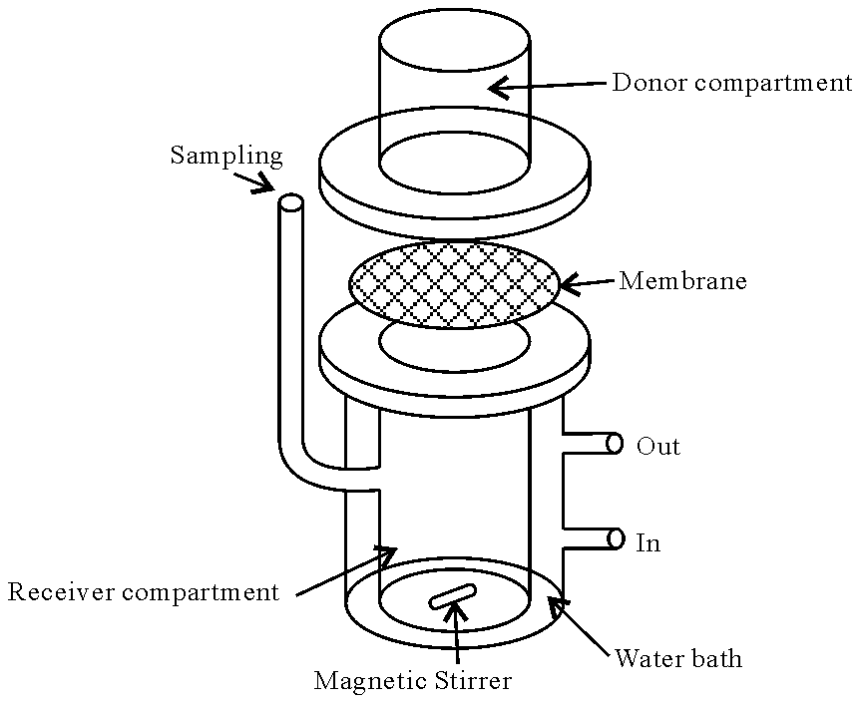
Modified Franz diffusion cell.

The skin permeation experimental protocol was approved by the Institutional Animal Care and Use Committee of Kaohsiung Medical University (Kaohsiung, Taiwan). The committee confirmed that the permeation experiment followed the guidelines as set forth by the Guide for Laboratory Fact lines and Care. The *in vitro* skin permeation of RHCl from nanoemulsion formulations and control groups determined using a modified transdermal Franz diffusion cell [31] (Fig. 1) with an effective diffusion area of the cell was 3.46 cm^2^ and receptor compartment volume of 20 mL. The abdominal skin of excised Wistar albino rat (275–300 g) was mounted on the receptor compartment with the stratum corneum side facing upward to the donor cell. The donor cell was loaded with 1 mL of samples and occluded by para film. The temperature of receiver vehicle of pH 7.4 phosphate buffer containing 40% PEG400 (drug solubility of 67.2 ±0.5 mg/mL) was maintained at 37±0.5 °C by thermostatic pump and was constantly stirred at 600 rpm by a magnetic stirrer during the experiment. At specific intervals, *i.e.*,1, 2, 3, 4, 5, 6, 8,10, and 12 h, one milliliter of receptor medium was withdrawn via the sampling port and was analyzed for drug content by modified HPLC method [32]. All experiments were repeated three times and averaged.

### *Chromatographic condition* HPLC analysis of RHCL

A Hitachi L-7100 series HPLC system and a LiChroCART RP-18e column (125×4 mm I.D., particle size 5 µm) were used for RHCl analysis. A mixture of 60% 0.05 M ammonium acetate buffer containing 0.05% triethylamine (adjusted to pH 7.0 by hydrochloride) and 40% methanol was used as mobile phase. The flow rate and detection wavelength were 1 mL/min and 250 nm respectively. Internal standard was caffeine of 100 µg/mL. The concentration of RHCl ranged from 3 to 200 µg/mL with a linearity of (r^2^ = 0.9998). The limit of quantitation was 1 µg/mL. The precision, as coefficient of variation (CV, %), was calculated for all the calibration standards. Accuracy was calculated as relative error (RE, %). The CV and RE values were less than 1.7% and 6.7% respectively.

### Data analysis

The cumulative amount of RHCl transported through rat skin was plotted as a function of time, and the linear regression analysis was used to determine the permeation rate (flux) of RHCl. The time of first-detected RHCl was set as lag time (LT). The formulation variables (X_1_, X_2_ and X_3_) and responses (flux and LT) of model RHCl-loaded nanoemulsions were analyzed by using Design-Expert^®^ software. Polynomial equations of linear, quadratic, and cubic forms were utilized to depict the relationship between independent variables and responses. The statistical parameters: the multiple correlation coefficient, the adjusted multiple correlation coefficient, the coefficient of variation, and the *p* value of model as well as lack of fit were used to confirm the suitable model equation for representing the relationship of formulation variables and responses.

### Stability

The RHCl-loaded nanoemulsion was stored in dark-brown bottles for protection from light. The stability of drug-loaded nanoemulsion formulation was evaluated via clarity and phase separation observation, and drug content at 25°C and 40°C.

## Results and Discussion

### Physicochemical characteristics of drug-loaded nanoemulsion

The mean droplet size and droplet size distribution (polydispersity index) and viscosity of experimental RHCl formulations are listed in Tables 1 and 2. The mean droplet size ranged from 14.5 to 107.9 nm, demonstrating all experimental nanoemulsion formulations were submicron emulsions. The polydispersity index ranged from 0.33 to 0.47, indicated a narrow deviation of average size. The viscosity of drug-loaded formulations ranged from 7.53 to 13.07×10^3^ cps at 37°C. It was found that the viscosity slightly increased when higher levels of surfactant were incorporated. A previous study pointed that the progress of emulsification is yielded by viscous liquid crystalline gel building at the interface between surfactant and water at high surfactant levels [33]. Nanoemulsions with higher levels of ethanol showed lower viscosity. The result might be attributed to the cosurfactant being able to decrease surface tension, and this then led to increased liquidity of the interfacial layer [19], [20]. In addition, it can be seen that the viscosity of nanoemulsion with IPA was lower than that of nanoemulsion with ethanol, demonstrating that cosurfactant type will influence the characteristics of the nanoemulsion [19], [20].

**Table 2:** The composition, physicochemical properties, and permeability parameters of model RHCl-loaded nanoemulsions provided mixture design.

	X_1_%	X_2_%	X_3_%	Size (nm)			PI			Viscosity (cps×10^3^)			Flux (µg/cm^2^/h)			LT (h)		
F01	20.0	30.0	44.5	12.1	±	1.1	0.36	±	0.07	12.73	±	0.21	33.04	±	2.72	4.0	±	0.6
F02	25.9	30.0	38.6	12.5	±	1.6	0.45	±	0.06	9.79	±	0.07	33.98	±	3.71	3.7	±	0.6
F03	20.0	30.0	44.5	12.6	±	0.2	0.41	±	0.06	12.67	±	0.06	33.76	±	4.54	4.0	±	1.0
F04	25.9	25.9	42.7	24.5	±	1.5	0.32	±	0.04	8.82	±	0.07	45.67	±	4.93	3.7	±	0.6
F05	30.0	30.0	34.5	53.2	±	7.3	0.57	±	0.12	8.52	±	0.02	35.53	±	2.31	3.7	±	0.6
F06	30.0	25.7	38.8	24.4	±	3.6	0.30	±	0.03	7.80	±	0.05	56.93	±	13.16	3.0	±	1.0
F07	30.0	30.0	34.5	55.5	±	3.8	0.61	±	0.03	8.60	±	0.11	41.23	±	5.91	3.7	±	0.6
F08	30.0	20.0	44.5	15.4	±	0.8	0.32	±	0.06	6.55	±	0.00	63.23	±	6.32	3.0	±	0.0
F09	24.5	20.0	50.0	16.1	±	0.8	0.26	±	0.05	7.75	±	0.03	59.91	±	5.30	2.7	±	0.6
F10	20.0	24.5	50.0	14.3	±	1.2	0.34	±	0.06	10.99	±	0.06	49.80	±	2.13	3.0	±	0.0

The amounts of RHCl and IPM in formulations were fixed at 0.5% and 5% respectively.

The total amount of three variables of X_1_ (isopropyl alcohol, 20∼30%), X_2_(mixture surfactant of Brij30/Brij35 at ratio of 4/1, 20∼30%), and X_3_ (distilled water, 34.5∼50.0%) was 95%. X_1_+X_2_+X_3_ = 0.95.

LT: lag time; PI: polydispersity index.

### Skin permeation study

**Figure 2:**
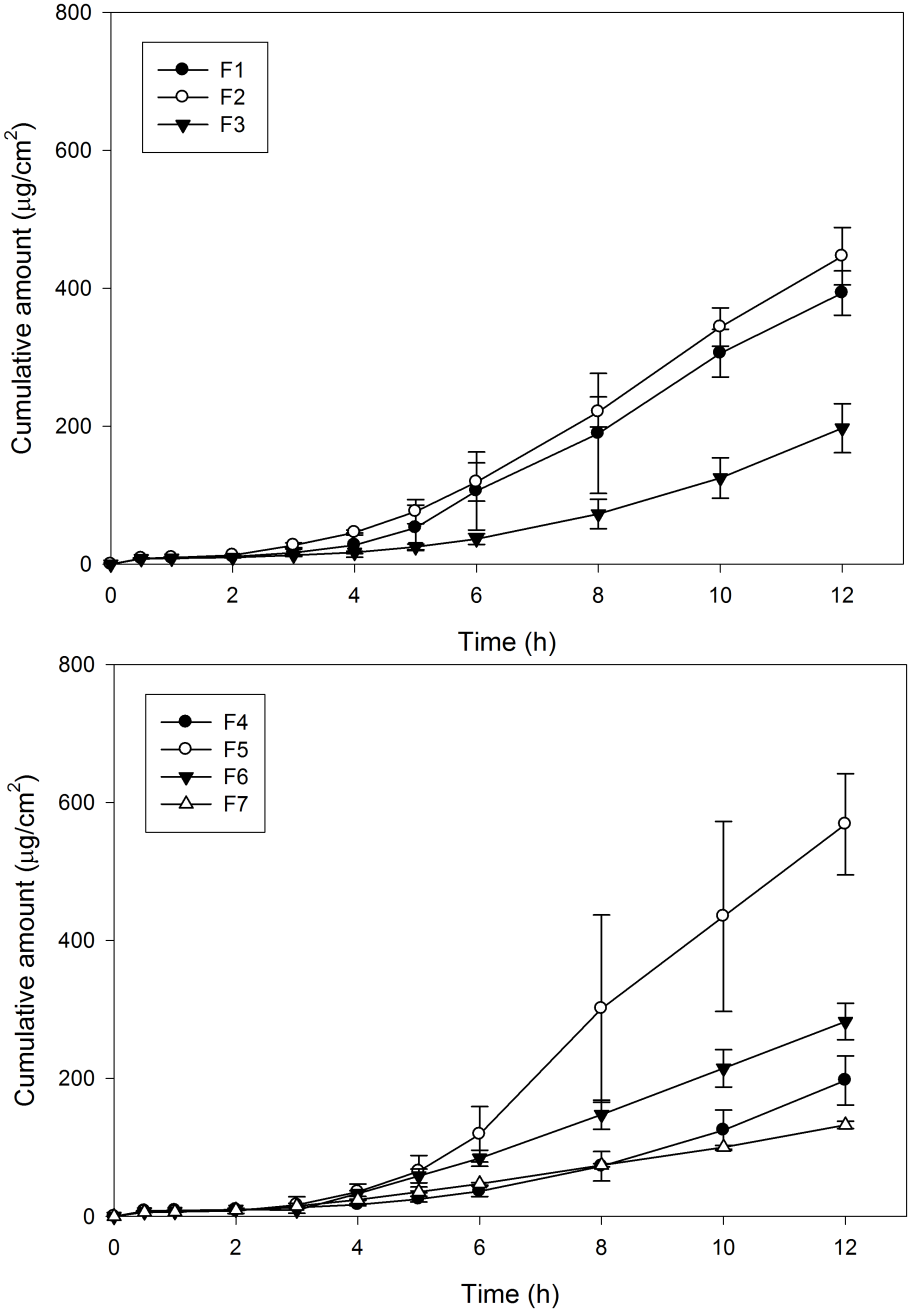
In vitro penetration-time profile of RCHl-loaded nanoemulsions with different combinations through rat skin. (n = 3).

The permeation profiles of RHCl-loaded nanoemulsions through the skin are plotted in Fig. 2. The permeation parameters including flux and lag time (LT) of RHCl-loaded nanoemulsions with different composition and proportion are listed in Table 1. The 2.5% RHCl of aqueous solution and aqueous solution containing 40% ethanol were used as control groups. In permeation study, no drug was detected at end time point of the experiment (12 h) indicating that the hydrophilic compound RHCl has difficulty in being transported through the skin barrier, even using 40% ethanol as a penetration enhancer. As shown in Table 1, when used nanoemulsion as the carrier vehicle, the permeability of the hydrophilic compound RHCl through skin was significantly improved. The result was in accordance with previous studies reporting that nanoemulsions could modify the surface electrical charge of an ionic drug and then enhance the permeability of a hydrophilic drug [34]–[37].

In comparing the effect of composition and proportion of formulation on the permeation capacity of the drug, it was found that flux increased by decrease in the amount of surfactant incorporated (F1∼F3). The result might be due to the thermodynamic activity of drug decreased and viscosity increased in formulation containing higher levels of surfactant [14]. When the level of ethanol in nanoemulsions increased from 20% to 30% (F3 vs F4, p>0.05), the flux slightly increased from 20.25 to 25.94 µg/cm^2^/h, and lag time decreased from 5.3 h to 4.3 h. Used IPA instead of ethanol as cosurfactant (F4 vs F6, F1 vs F5), the viscosity decreased and flux increased. Therefore, IPA was used in the follow-up experiment. In using mixed surfactants of Tween80/Span20 instead of mixture surfactant of Brij30/Brij35 (F1 vs F7, p<0.05), the flux of drug decreased, hence the mixture surfactant of Brij30/Brij35 was chosen as surfactant for subsequent experiments.

In order to evaluate the degree of effect of each component and the interaction components of nanoemulsion formulation on the permeation capacity of the drug and to acquire an optimal formulation, response surface methodology [28]–[30] was used in the present study. According to the above result, the oil amount was fixed at 5%, while the other ingredients of cosurfactant of IPA, mixture surfactant of B30/B35 and distilled water were set as variable factors and range of 20∼30%, and 20∼30% and 34.5∼50% respectively in this study. Ten model 0.5% RHCl-loaded nanoemulsions were prepared based on the mixture design provided by Design-Expert^®^ software. The permeation parameters of the drug were estimated by in vitro permeation study. The permeation parameters of all RHCl-loaded nanoemulsions are summarized in Table 2. The flux and LT of RHCl-loaded nanoemulsions ranged from 33.04 to 63.23 µg/cm^2^/h and 2.7 to 4.0 h respectively, indicating that the permeability of RHCl from nanoemulsions was significantly influenced by the composition proportion of formulations.

The flux and LT of RHCl-loaded nanoemulsions were set as responses, and the level of component of formulations set as variable factors were statistically analyzed using the RSM provided Design-Expert software.

The polynomial equation to depict the flux may be indicated thus: Flux = 1.64X_1_-1.55X_2_+1.05X_3._

**Figure 3:**
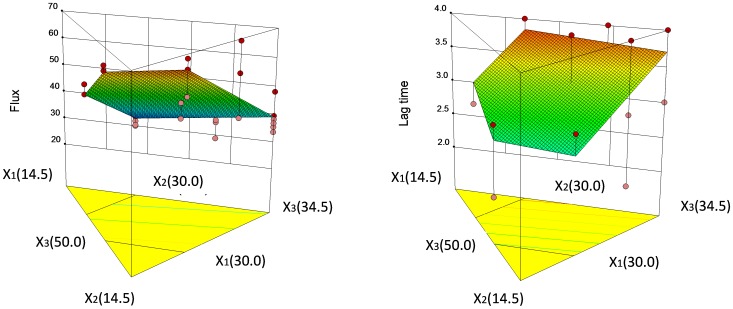
Three dimensional response surface plots illustrating the effect of IPA (X_1_), mixture surfactant (X_2_) and distilled water (X_3_) on the flux and lag time (LT) of RCHl-loaded nanoemulsions.

The *p*-value of the model polynomial equation was less than 0.001, demonstrating that the model was adequate to describe the relationship between independent and dependent variables. The *p*-value of the lack of fit was 0.2909, revealing no indication of significance, and further verified the satisfactory fitness of the model. The coefficients value of X term presented the effect degree of the independent factors on the dependent factors (responses). A positive sign displays a synergistic effect while a negative term shows an antagonistic effect on the dependent factors. The response surface plots illustrating the simultaneous effect of the independent factors on dependent variables (Flux and LT) are represented in Fig 3. The result showed that the IPA (X_1_) and mixture surfactant (X_2_) had similar effect on the drug permeation rate, followed by aqueous phase (X_3_). The flux increased with an increasing level of IPA and water and a decreasing level of mixture surfactant (Fig. 3).

To describe the LT, the mathematical polynomial equation might be written thus: LT = -0.016X_1_+0.108X_2_+0.011X_3._

The *p*-values of the model and lack of fit were <0.0001 and 0.4969 respectively, which demonstrated that the mathematical polynomial equation can describe the relationship between the formulation variables and LT. The three dimensional surface plot was graphed according to the mathematical polynomial equation and is shown in Fig. 3. It was found that the surfactant (X_2_) showed the greatest effect, followed by cosurfactant (X_1_) and distilled water (X_3_). The cosurfactant showed that the negative effect indicated lag time could be shortened by the increase in the level of IPA. The reason might be attributed to the cosurfactant decreasing the viscosity, and this then led to the increased diffusivity of the drug (reduction in the lag time) [19]. To validate the predictive ability of the hypothesized mathematical model, an optimal nanoemulsion with flux and LT values of 57.90 µg/cm^2^/h and 2.98 h respectively, when level of X_1_, X_2_ and X_3_ were 30%, 22.7% and 41.8% respectively was predicted by the response surface methodology. A new RHCl-loaded nanoemulsion was prepared and obtained flux as well as LT values of 58.55±5.75 µg/cm^2^/h and 3.0±0.0 h respectively. The predicted and observed values showed no significant difference, indicating that the response surface methodology can be used to design RHCl-loaded nanoemulsions.

### Stability

After 3 months storage at 25°C and 40°C, the apparent RHCl-loaded nanoemulsion had no obvious change, and no drug crystal was observed. After storage, mean droplet size showed non-significant change, from 19.0±5.0 nm to 22.3±3.1 nm for 25°C storage and 23.5±4.2 nm for 40°C storage. The residual drug contents tested drug-loaded nanoemulsions at 25°C and 40°C storage were 98.89±3.90% and 98.46±2.56% respectively, indicated that RHCl-loaded nanoemulsion was stable.

## Conclusions

The permeation rate of hydrophilic compound increased from 0 to 63.23 µg/cm^2^/h and the lag times were also shortened from more than 12 h to 2.7 h by using nanoemulsions as carrier vehicle, suggesting a promising role of nanoemulsions in enhancing the permeability of RHCl. An appropriate combination and proportion of nanoemulsion formulation including the type and level of oil, surfactant, cosurfactant, and water is a major consideration for the transdermal drug delivery.
